# Molecular Communication of a Dying Neuron in Stroke

**DOI:** 10.3390/ijms19092834

**Published:** 2018-09-19

**Authors:** Berta Puig, Santra Brenna, Tim Magnus

**Affiliations:** Experimental Research in Stroke and Inflammation (ERSI) Group, Department of Neurology, University Medical Center Hamburg-Eppendorf, 20246 Hamburg, Germany; s.brenna@uke.de

**Keywords:** stroke, brain ischemia, neuronal cell death, apoptotic bodies, synapses, extracellular vesicles

## Abstract

When a main artery of the brain occludes, a cellular response involving multiple cell types follows. Cells directly affected by the lack of glucose and oxygen in the neuronal core die by necrosis. In the periphery surrounding the ischemic core (the so-called penumbra) neurons, astrocytes, microglia, oligodendrocytes, pericytes, and endothelial cells react to detrimental factors such as excitotoxicity, oxidative stress, and inflammation in different ways. The fate of the neurons in this area is multifactorial, and communication between all the players is important for survival. This review focuses on the latest research relating to synaptic loss and the release of apoptotic bodies and other extracellular vesicles for cellular communication in stroke. We also point out possible treatment options related to increasing neuronal survival and regeneration in the penumbra.

## 1. Introduction

Stroke is the second biggest cause of death worldwide and has maintained its status as one of the biggest killers for the last 15 years (http://www.who.int/en/news-room/fact-sheets/detail/the-top-10-causes-of-death). It is also the third biggest cause of disability worldwide, representing a high economic and social burden for communities. Although the incidence, prevalence, and mortality of stroke has tended to decrease in recent years, the absolute number of people affected is increasing, especially in low- and middle-income countries [1].

Ischemic stroke by blockage of a brain artery accounts for 87% of the cases. Following transient or permanent lack of blood flow—and thus of glucose and oxygen supply to the brain—the area with severe hypoperfusion is known as the core of the infarct where neurons are lethally injured. This area is variably surrounded by a less hypoperfused area (known as the penumbra) where cells are still metabolically active for a certain period and—depending on circumstances—will either die or survive [2].

Neuronal cell death in stroke is multifactorial and complex [3], comprising several components that contribute to excitotoxicity, oxidative stress, mitochondrial dysfunction, and neuroinflammation. To find mechanisms of neuroprotection for this area is an active focus of research [4]. At present, however, the only therapeutic options are local recanalization and systemic thrombolysis, which have a short therapeutic window, with only 20% of patients eligible for these treatments [5].

Importantly, the amount of permanent damage is proportional to the duration of ischemia; therefore, to restore the blood flow as soon as possible is fundamental [6]. Paradoxically, the necessary reperfusion also contributes to generate reactive oxygen species (ROS) and nitrosylation, which in turn activates the immunological response, leading to neuroinflammation with detrimental consequences [7]. During neuroinflammation, the first cells to react to the ischemic injury are microglia cells (i.e., the resident immune cells of the brain), and the immune response is followed by infiltration of macrophages, lymphocytes, neutrophils, and dendritic cells to the ischemic parenchyma due to blood–brain barrier breakdown, which exacerbates the damage [8]. Neuroinflammation may also play an essential role in brain damage and brain repair [9,10,11,12]. 

Neurons, due to their inherent high demand of energy, are very sensitive to the lack of glucose and ATP and are the first brain cells to die in the area directly affected by the lack of blood flow [13]. Loss of neurons can continue for hours or even days after reperfusion, depending on the cellular characteristics of the area affected [14].

Neuronal cell death is not an isolated process, implicating a full response from various brain cells. On the one hand, neurons are connected to each other forming an extensive communication network through synaptic transmission. Failure in the synaptic process causes disconnection and transsynaptic degeneration, leading to neuronal dysfunction and cell death to neurons that are in related cerebral structures [15]. On the other hand, we are starting to recognize important intimate interactions between all brain cells, such as the relationship between glial cells, neurons, and blood vessels in the so-called neurovascular unit (NVU). For example, the NVU regulates not only the cerebral blood flow according to the energy needs of the brain but also has a significant function in maintaining the blood–brain barrier [16,17]. To maintain these structures, communication between cells is a key process.

The concept of neuronal communication has also evolved in recent years. In 1980, Barker et al. suggested that other types of neuronal communication (apart from synaptic transmission) have to exist to explain the effects that peptides were producing to the excitability pattern [18], defining the terms “neurohormonal communication” and “neuromodulation”. The picture today has become more complicated with the recognition of extracellular vesicles (EVs), electrical synapses, and tunneling nanotubes [19,20,21,22].

This review summarizes the current knowledge on how dying neurons influence other brain cells through synaptic loss and EVs—two crucial processes influencing ischemic damage with therapeutic implications. On the one hand, due to high energetic demands, decreased synaptic strength and synaptic loss occur before neuronal death in the penumbra and is probably one of its causes. On the other hand, EVs are strong communication tools implicated in inflammation, cancer progression, and neurodegeneration that can be released from all brain types. EVs contain proteins, mRNA, miRNA, and lipids that are able to modify the behavior of the recipient cells in healthy and diseased conditions [23]. For example, in Alzheimer’s disease, EVs are implicated in the spreading of amyloid proteins and toxicity [24,25]. We will discuss their role in ischemia, and we will try to define mechanisms that are potentially useful as therapeutic targets.

## 2. Cell Death in Stroke

Cell death in stroke has been extensively reviewed [26,27]. In short, when the blood flow supply is decreased to less than 20%, the brain tissue becomes severely damaged. The hypoxia leads to ATP depletion in a matter of minutes, inducing the failure of the Na+/K+ pump, neuronal depolarization, and release of glutamate. Because glutamate cannot be taken up by either neurons or astrocytes [28], levels of glutamate rapidly rise in the extracellular space, leading to activation of glutamate receptors (mainly *N*-Methyl-d-Aspartat Receptors, NMDAR) that allow the entrance of Ca^2+^. Increased intracellular Ca^2+^ activates calpains and phospholipases, leading to excitotoxic cell death. Several types of cell death are linked to excitotoxicity, such as apoptosis, oncosis, autophagy, ferroptosis, and phagoptosis [27].

Cells in the ischemic core die from oncosis, accidental cell death due to rapid depletion of intracellular ATP, impairment of the ionic pumps, and rapid increase in intracellular Ca^2+^. Oncosis is characterized by swelling of the organelles, leading to plasma membrane disruption and cell death. The term necrosis is sometimes used as a synonym of oncosis; in other cases, necrosis is considered as the postmortem morphological changes after oncosis (defined in biochemical terms) [27,29,30]. In any case, because there is a disruption of the plasma membrane, it provokes tissue inflammation [31]. The massive entrance of Ca^2+^ activates the neuronal nitric oxide synthase (nNOS) and calpain I, producing an increase in reactive oxygen species (ROS). The massive entrance of Ca^2+^ causes mitochondrial membrane depolarization and the opening of the mitochondrial permeability transition pore (mPTP), causing mitochondrial permeability transition, an increase in ROS, and cell death, usually by necrosis [32]. In addition, necrosis after ischemia can also be triggered in a regulated fashion (necroptosis) mediated by TNFα/FAS and RIPK1-RIPK3 signaling cascade [33,34] and probably also through AKT and mTOR pathways [35]. This fact opens the possibility that the necrotic core can be therapeutically targeted [36,37,38,39].

The “penumbra” marks an area where cells have no electric activity but are still metabolically active. Because they will receive deleterious signals from the dead cells of the ischemic core in the following hours, they will perish within a few days [26], aggravating ischemic damage and the clinical outcome [40]. As this area could be potentially rescued, it has been an intense field of research for the last 40 years [41,42]. In the penumbra—by the fact that cells still have energy in the form of ATP—cells are usually able to die in a regulated manner by programmed cell death (PCD), which has the advantage that the internal content is not released to the extracellular milieu, thus sparing detrimental inflammatory processes [43,44,45,46]. PCD in the ischemic penumbra can be mediated by apoptosis or autophagocytosis or a mixture of both [47], probably following distinct temporal dynamics [48]. However, if apoptotic cells are not promptly cleared, apoptosis can lead to secondary necrosis and then trigger inflammation [31,49].

In the next sections, we will focus on what happens to neurons in the penumbra from lack of electrical impulses to death and how these dying neurons communicate to other cells in the brain. We will pay particular attention to EVs.

## 3. Loss of Synapses in the Penumbra: From Synaptic Failure to the Fatal Commitment Point

The brain is a high energy consumer—it constitutes 2% of body mass but uses 20% of all oxygen consumed by the body [50]. The synapse is the site where most energy will be spent. Thus, in the rat brain, the process of releasing one vesicle of glutamate (consisting of bringing the vesicle to the presynaptic membrane, releasing it, and recycling of the vesicle and glutamate by the astrocyte) uses up around 1.64 × 10^5^ ATP molecules. The energy expenditure for action potentials and postsynaptic potentials is even higher [51]. In humans—with fewer neurons per mm^2^ but more synapses—it is calculated that the consumption is approximately three times bigger [52]. The main source of ATP in the brain is generated from the complete glucose oxidation in the mitochondria, a process that also takes place at the synaptic level. Neurons take up glucose from the extracellular milieu, but astrocytes can also deliver substrates for oxidation (glutamine, lactate, or ketone bodies) to neurons [53,54,55]. Due to the high energy need, the lack of glucose and oxygen derived from transient ischemia represents a big problem for synapses, which can lead to neuronal death.

As stated above, neurons at the penumbra are structurally conserved and metabolically active; however, due to the low amounts of ATP, they enter into electric silence as a consequence of synaptic failure [56]. This electric silence could be either a mechanism to save energy to survive until homeostatic conditions are restored [57] or can be a consequence of synaptic modifications. Thus, synaptic failure could occur due to presynaptic alterations, such as changes in Ca^2+^-dependent processes or deficient synapsis phosphorylation, leading to impaired docking to the presynaptic membrane [58,59]. It could also be due to postsynaptic failure, probably as a consequence of Ca^2+^ entrance [60,61]. Recent studies have pointed out that lack of proper vesicle endocytosis and decreased exocytosis account for the synaptic silence in rat neurons subjected to an in vitro model of hypoxia, thus indicating that electric silence is a consequence of presynaptic compartment modifications [62]. Moreover, overactivation of the NMDA receptors due to excitotoxicity leads to alteration in synaptic proteins localization, probably due to cytoskeleton disassembly, translation shutdown, decreased endocytosis, and activation of calpains. The final consequence is synaptic disruption and neuronal loss [63].

At the point of synaptic failure, neurons can either recover or go to apoptosis. In an in vitro model using rat primary neurons subjected to lack of oxygen (simulating ischemic penumbra), leFeber et al. recently showed that neurons, although losing synaptic connectivity, can potentially recover from hypoxic conditions when reoxygenated up to 12 h. In the period between 6 and 18 h, they can even partially recover activity, probably as a compensatory mechanism. After this period, neurons decrease their electric activity reaching a “point of no return” (commitment point) after 30–40 h of hypoxia. After reaching this point, neuronal death is independent of the hypoxic depth applied to the cells but related to the missing connectivity [64]. Therefore, the fact that synaptic connectivity plays a central role in the fate of a neuron after ischemic conditions (at least in vitro) needs to be taken into consideration. In a permanent model of focal middle cerebral artery occlusion (pMCAO) in mice, it has been shown that astrocytes and microglia upregulate SPARC (secreted protein, acidic, and rich in cysteine) helping to increase synaptic strength, probably as an attempt to preserve neuronal connections after injury. Likewise, neuronal cultures subjected to hypoxia conditions pretreated two days earlier with recombinant SPARC showed reduced synaptic loss and increased survival [65].

Another way of miscommunication in the peri-infarct area is the spreading depolarizations (SD). Neurons at the necrotic core depolarize due to anoxia, releasing glutamate and K^+^. Neurons at the penumbra also depolarize, but because their energy depletion is not that acute, they can repolarize by using ATP-dependent membrane pumps. However, because they receive glutamate and K^+^ from the necrotic core, they depolarize again. Thus, SDs spread from affected tissue to unaffected tissue with repetitive sequences of depolarization/repolarization, which can last for hours and further depletes the neurons of energy. In the rat model of ischemia (transient middle cerebral artery occlusion, tMCAO), SDs at the penumbra (peri-infarcts depolarizations, PID) is one of the major factors that leads to neuronal death [66]. The outcome of the neurons at the penumbra suffering from PIDs depends on distance to the noxious condition, the specific neuronal population, the anatomical structure, and the time of reperfusion [67,68].

## 4. Signals from Apoptotic Neurons: Who Is Cleaning the Mess?

**Figure 1:**
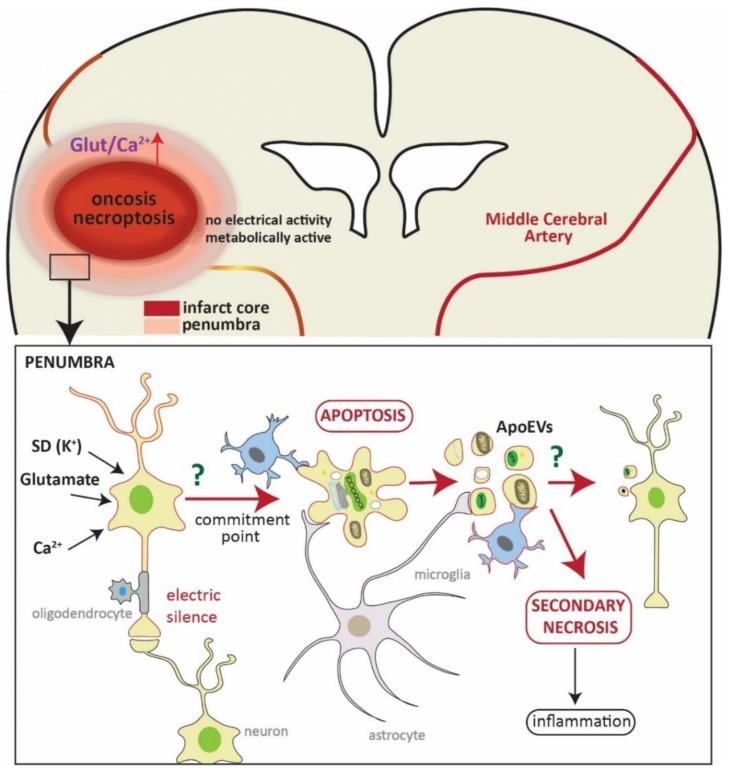
Schematic representation of neuronal death in the penumbra. In the upper part, representation of a coronal section of the brain where a brain artery (middle cerebral artery) is blocked (yellowish line). The hypoperfused area (core of the infarct), in which cells eventually die by oncosis and necroptosis, is shown in red. This area is variably surrounded by a less affected area—the penumbra. The square refers to a magnification of this area (shown in the lower part). Here, neurons are affected by an influx of glutamate, Ca^2+^, and spreading depolarizations (SD), which are deleterious signals from the core of the infarct. These neurons are metabolically active but do not present electrical activity due to synaptic failure (electric silence). At this point, neurons can either die by apoptosis or recover, and the fate of neurons depends on many factors, such as distance to the core of the infarct, time of reperfusion, and neuronal population. Once the neuron gets to the commitment point, it will die by apoptosis (and/or autophagocytosis). Cells will then be engulfed by microglia (also by astrocytes and macrophages) without eliciting inflammation. One characteristic of apoptotic cell death is the formation of apoptotic bodies (apoptotic extracellular vesicles, ApoEVs) which are easy to discard. It has recently been shown that the content of ApoEVs is not random, and cells can actively sort DNA and selected proteins into them, thus bestowing ApoEVs a role in communication. Whether microglia or neurons show a specific reaction to ApoEVs in stroke is unknown to date. The microglia cell framed in red represents a hypothetical reaction to the ApoEVs as it has been observed in glioblastoma [92,93]. If ApoEVs and apoptotic cells are not properly cleared, they trigger secondary necrosis, eliciting inflammation.

Once neurons in the penumbra get to the commitment point, they mainly die by apoptosis (with possible participation of autophagy-like cell death (Figure 1) [27,47]). Apoptosis is characterized by a defined pattern of morphological and biochemical changes. Apoptotic cells or bodies are then usually engulfed by phagocytes without eliciting any immunoreaction [69,70]. Apoptotic neuronal cells are cleared by microglia, the resident myeloid-lineage derived cells of the brain [71,72,73]. Microglia recognize “find me” signals such as released fractalkine/CX3CR1 [74], “eat me” signals such as plasma membrane exposition of phosphatidylserine (PS) or calreticulin [75], and the loss of “don’t eat me” signals such as CD47 or polysialylated proteins [76] from apoptotic cells (reviewed in References [77,78]). They are capable of engulfing whole neurons, synapsis, and cell debris. However, under inflammatory conditions, activated microglia release peroxynitrite, which provokes neurons to transiently—but reversibly—expose PS. This process is known as phagoptosis, where microglia can phagocyte stressed but “alive” neurons [79,80,81,82]. Phagoptosis is a mechanism that contributes to delayed neuronal loss in an animal model of focal ischemia, and inhibiting this process can reduce infarct volume, indicating that the neurons otherwise eliminated by phagoptosis are fully functional [83].

It has been recently observed that astrocytes can also behave as nonprofessional phagocytic cells [84,85], and they can contribute to the elimination of apoptotic neurons, synapses, and cell debris after transient focal ischemia in mice [86]. There is a distinct spatiotemporal pattern of astrocyte phagocytosis in comparison to microglia, with astrocytes present at the penumbra with a delayed time onset, probably helping area remodeling and recovery. The same observation was recently made in a mouse model and in human brain samples of Alzheimer’s disease, where astrocytes were engulfing (although with low efficiency) plaque-associated dystrophic neuritis, indicating a probable common process of neurodegeneration [87].

A new concept has been recently proposed, namely, that neurons that suffer from mild excitotoxic damage are also capable of releasing “help me” signals to get assistance from other cell types, principally microglia and vascular cells. These “help me” signals comprise cytokines, chemokines, and growth factors or even molecules related to “find me” and “eat me” signals. The release of these signals from injured but alive neurons can induce a beneficial microglia phenotype, participating in neuronal recovery by releasing neurotrophic factors [88]. These signals are different from the danger-associated molecular pattern (DAMPs such as ATP, high mobility group box 1 (HMGB1) and heat-shock proteins), which are also released from dying neurons, that trigger the inflammatory response [89]. How the combination of all these signals influences the outcome of the ischemic injury is at present not known.

The inflammatory response following ischemia is accompanied by the infiltration of monocytes to the brain parenchyma [8,89]. Recruited monocytes become macrophages, helping with debris clearance, although it seems that microglia-derived macrophages are more effective [90,91].

## 5. Apoptotic Bodies: More Than Silent Clearing Vesicles?

Cells that die by apoptosis follow a defined morphological pattern that is characterized by cell shrinkage and pyknosis due to chromatin condensation. Moreover, the plasma membrane protrudes (apoptotic membrane blebbing) generating apoptotic bodies (ApoBD)—broadly defined as apoptotic extracellular vesicles (ApoEVs) depending on the size, ranging from 0.8 to 5 µm [94]—that engulf cytoplasm and well-preserved organelles [69,95]. Not all cells that undergo apoptosis can produce ApoEVs but in the case of neurons, it has been shown that they produce ApoEVs, which are cleared by microglia [73,96]. The formation of ApoEVs is complex and comprises several steps, and ApoEVs are much more efficiently cleared by macrophages or neighboring cells than whole apoptotic neurons [97]. Nevertheless, not all ApoEVs are effectively removed. In 1972, it was observed that small ApoEVs are dispersed and seen in the extracellular space [69]. More recently, it was observed that a type of ApoEVs generated by monocytes through a process known as “beaded apoptopodia” actively sort proteins implicated in signal transduction, cell growth, maintenance, transport, and kinases to ApoEVs, whereas nuclear proteins are actively excluded [94]. Thus, the fact that some ApoEVs are not rapidly eliminated and that the content is actively selected leads to the idea that they might have a function beyond the elimination of apoptotic cells [98]. Holmgren and colleagues showed in 1999 that DNA could be transferred from one cell to another through ApoEVs [99]. More support for the idea that ApoEVs have a role in transferring information comes from the cancer field [100,101], where it has been observed that cancers with a high rate of apoptosis are the most aggressive, which on first glance would be counterintuitive [102]. An explanation for this fact has very recently come from a study by Pavlyukov et al. They could show that highly malignant, therapy-resistant glioblastomas upregulate and release splicing factors through ApoEVs that transfer therapy resistance to neighboring surviving cells [103]. Therefore, it is likely that even in stroke, apoptotic cells communicate through ApoEVs with their neighboring cells.

However, as for apoptotic cells, the suboptimal clearance of ApoEVs leads to secondary necrosis, resulting in inflammation [104]. Thus, apoptosis induced in endothelial cells by serum deprivation and TNFα exposition triggers the in vitro release of ApoEVs. The intraperitoneally injection of these ApoEVs in mice causes neutrophilic inflammation mediated by IL1α, which is present in the ApoEVs [104]. There seems to be a dual role for ApoEVs depending on their content, size, and time of clearance.

## 6. Role of Other Extracellular Vesicles (EVs) in Ischemic Damage

ApoEVs are the less-studied vesicles from the group collectively known as extracellular vesicles (EVs). This group comprises, at least, ApoEVs, exosomes, and microvesicles (or ectosomes), which have been recognized in the last two decades as an emergent, important communication tool between cells [23,105]. All brain cells can release EVs, including neurons, glia, endothelial cells, and pericytes in healthy and in diseased conditions [106,107]. The three types of vesicles differentiate themselves by size, origin, and content. ApoEVs range between 0.8 and 5 µm and originate at the plasma membrane from neurons undergoing apoptosis. Exosomes—ranging approximately between 30 and 150 nm—are originated inside the cell as vesicles formed in the multivesicular endosomes (MVE) and therefore have specific markers of late (some also early) endosomes (such as CD63, Rab7, and Alix). Microvesicles ranging from 100 nm to 1 µm originate from direct budding of the plasma membrane. The boundaries between one type of vesicle to another are still confusing, but a big effort is being made to categorize and differentiate the diversity of EVs [108]. Hence, some studies have shown that different types of vesicles differ in RNA and protein cargo [109,110] and even the same cells can deliver exosomes differing in composition, leading to diverse effects in recipient cells [111]. As for ApoEVs, exosomes were first described as vehicles to discard unwanted components from the cell (e.g., to discard transferrin receptor in the maturation process of reticulocytes to erythrocytes [112]). However, more recently, specific functions have been attributed to them [113].

Most of the variability and comparability between studies comes from the method of preparation and from the fact that our knowledge about function and composition is constantly changing and evolving [106,108,114].

**Figure 2:**
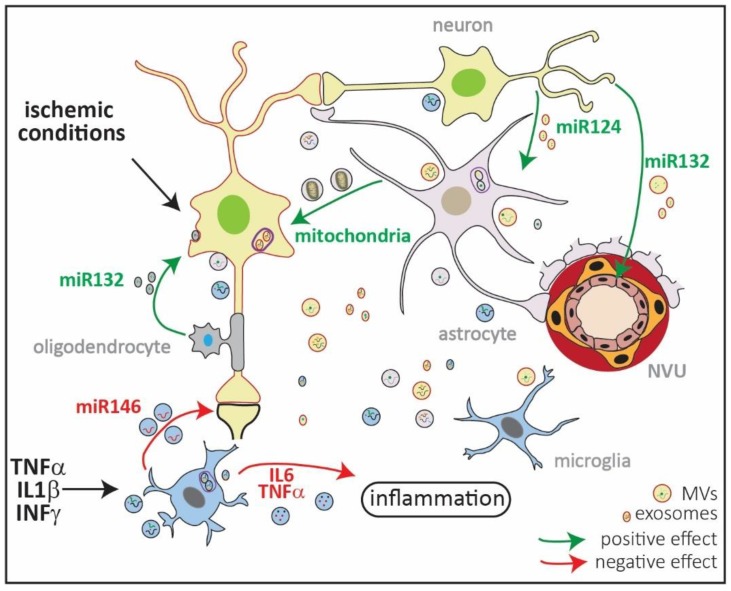
Schematic representation of extracellular vesicles (EVs) in ischemic injury. EVs comprise microvesicles (MVs) ranging from 0.1–1 µm, exosomes ranging from 30–150 nm, and ApoEVs ranging from 0.8–5 µm (Figure 1). They all have a different cellular origin, with MVs shed from the plasma membrane, while exosomes are formed in multivesicular endosomes (MVEs; in violet). All types of brain cells produce exosomes and MVs, which transport lipids, DNA, mRNA, miRNA, and proteins loaded in a stimulus-dependent manner. Under in vitro ischemic conditions, exosomes released by oligodendrocytes contain miR132 which are taken up by neurons and promote their survival [92,93]. By contrast, EVs released by microglia previously exposed to lipopolysaccharide (LPS) increase in size and content of IL6 and TNFα, thus exacerbating inflammation [92,93]. Microglia exposed to proinflammatory cytokines increase the release of miR146 via exosomes that are taken up by neurons, downregulating synaptotagmin 1 and neuroligin 1 and contributing to loss of excitatory synapsis [92,93]. Neurons can contribute to endothelial integrity and, thus, to the neurovascular unit (NVU) functionality by releasing miR132 in EVs. Neurons can also regulate the expression of GLT1 in astrocytes through vesicular miR124 [92,93]. Finally, it has been shown that astrocytes can release mitochondria in MVs, which contributes to neuronal survival in in vitro and in vivo models of ischemia [92,93]. The content of the exosomes and MVs in the picture is random and only depicts the variety of EV cargos.

EVs are present in biofluids, such as blood, saliva, urine, and the cerebrospinal fluid and are therefore very attractive biomarkers for diseases, including brain diseases [115]. Because of their importance and novelty, EVs communication in the brain has been extensively reviewed [23,105,107,116,117]. Therefore, we will focus on the possible role of EVs in ischemic damage and how they contribute to disease outcome (Figure 2).

EVs are capable of transporting proteins, lipids, DNA, mRNA, and noncoding RNAs such as miRNA, thus bestowing the capacity of modifying gene expression in distant cells [118]. Most of the research done in EVs regarding neuronal ischemia is related to the capacity of EVs released from mesenchymal stem cells (MSCs) as a therapeutic tool for stroke recovery or as potential biomarkers [119,120,121,122]. By contrast, the role of EVs from astrocytes, microglia, oligodendrocytes, or endothelial cells after ischemic insult has been scarcely studied [123]. Nevertheless, there are some studies that directly or indirectly point out to a possible contribution of EVs to ischemic damage. For example, it has been shown that oligodendrocytes can secrete exosomes after glutamate activation of NMDAR during neuronal activity and that neurons take up these exosomes. When neurons are subjected to metabolic stress or in vitro ischemic conditions (e.g., oxygen–glucose deprivation, OGD), incubation with oligodendroglial exosomes promotes cell survival [92,93].

After in vitro exposure to lipopolysaccharide (LPS), microglia change the composition, number, and size of EVs, which increase the vesicular release of IL6 and TNFα, exacerbating inflammation [124]. Microglia exposed to IL1β, TNα, and IFNγ cytokines secrete EVs that strongly differ in composition. When EVs from microglia exposed to these proinflammatory conditions are incubated with primary neurons in cell culture, there is a decrease in synaptic density and dendritic spines as a consequence of the downregulation of synaptic proteins through exosomal miR146. This suggests a link between chronic inflammation and cognitive deficits mediated by EVs [125].

As for potential biomarkers, miR9 and miR124 in EVs are increased in plasma serum of stroke patients, positively correlating with the degree of damage [126]. There is also an increase in miR223 in EVs, correlating with severity of the ischemic injury [127].

Importantly, EVs are also capable of transferring organelles, such as mitochondria. Recently, it was shown that astrocytes can transfer functional mitochondria through EVs to neurons. When neurons were subjected to OGD in vitro, incubation with conditioned media from astrocytes containing vesicles with mitochondria led to enhanced neuronal survival compared to neurons incubated with media depleted from these vesicles. Moreover, mice subjected to tMCAO and injected with conditioned media of astrocytes with labeled mitochondria showed increased neuronal survival in the penumbra, with neurons having incorporated these labeled mitochondria [128]. Thus, transfer of mitochondria from astrocytes to neurons through EVs could be a mechanism through which astrocytes not only help in the acute phase of stroke (neuronal survival) but could also participate in neuronal plasticity. 

The fact that the release of exosomes from neurons depends on Ca^2+^ and is regulated by synaptic activity [129] makes it likely that exosome release would increase in the penumbra of an ischemic area. For example, it has been observed that neurons can secrete exosomes containing miR124a, increasing the amount of glutamate transporter 1 (GLT1) expression and glutamate uptake by astrocytes. The fact that miR124 increases significantly in plasma of stroke patients shows a probable neuronal reaction to increased levels of glutamate. Moreover, neurons also influence the integrity of the NVU by releasing exosomal miR132 to be taken up by endothelial cells. Endothelial cells take up miR132, increasing vascular endothelial cadherin, an important protein present in the adherens junction [130]. Whether this has an impact after ischemic injury and the relevance of EVs secreted from the NVU after stroke is still unexplored.

Last but not least, EVs can also be isolated from brain, as has been shown for animal models of Alzheimer’s disease and Parkinson’s disease [131,132,133]. In these diseases, EVs play an important role in the spreading of misfolded proteins through the brain [24,131,134,135]. In a recent traumatic brain injury (TBI) study in mice, EVs isolated from brain showed an increase in several miRNAs, most prominently miR21. EVs-associated miR21 was released by neurons, probably mediating signaling from neurons to glia. Because miR21 has a neuroprotective role in an in vitro model of ischemia, it would be interesting to check if miR21 associated to EVs is also increased in animal models of stroke [136]. Thus, isolation of brain EVs opens a new line of research that will hopefully shed light on mechanism and therapies for diseases such as stroke.

## 7. Therapeutic Options to Salvage Neurons in the Penumbra

Because stroke is a disease where multiple brain cells are affected and where inflammation plays a fundamental role, a multitargeted therapeutic approach will probably be needed to minimize the consequences of ischemia and contribute to regeneration. Several reviews have focused on new therapeutic targets, ranging from inhibiting of excitotoxicity and related signaling pathways [137] to modulating the immunological response and to increasing cell clearance after apoptosis [138].

We would like to point out possibilities of treatment regarding synaptic protection and the use of EVs as therapeutic tools.

It has been shown that statins (such as simvastatin or atorvastatin), which are drugs used against hypercholesterolemia, can have beneficial effects in stroke by not only decreasing inflammation, oxidative stress, and improving the endothelial function but also by restoring proteins related to neuronal connectivity [63,139]. Similarly, new migraine treatments influence SD by targeting ion channels or neuromodulation [140] or by trying to control neuronal polarization using electrical fields [141]. In a combinatory approach, some of these treatment options might prove helpful in stroke.

It has been shown that the transplantation of mesenchymal stem cells (MSC) or neuronal progenitor cells (NPC) can reduce ischemic injury and infarct volume, and it seems likely that EVs exert these positive effects [121,142]. Recent experiments demonstrate that a single intravenous delivery of MSCs-derived extracellular vesicles (MSCs-EVs) to mouse subjected to subcortical stroke, show better functional recovery with increased myelinization and axonal sprouting compared to noninjected mice [143]. In another study, increased neurogenesis and angiogenesis was observed 28 days after MCAO in mice after intravenous delivery of MSCs-EVs [144]. At present, all the preclinical studies in rat or mice models of stroke injected with MSCs-EVs show functional recovery and brain repair [145], thus offering the possibility of a promising therapy. In humans, a patient presenting Graft-versus-Host disease (GvHD) treated systemically with exosomes presented an improvement in his condition, and the treatment was well tolerated [146]. However, because there are not yet standardized techniques for EVs preparations and quality controls, several precautions have to be taken into account to move to clinical trials with MSCs-EVs [147]. To our knowledge, a clinical trial to treat stroke patients with MSCs-EVs transfected with miR124 is in the database of the NIH clinical trials (http://www.clinicaltrial.gov (NCT03384433)), and it is estimated to start at the end of 2018.

EVs could also be used as vehicles by loading them with molecules that can then be precisely delivered to target cells [148]. EVs are ideal tools because they can cross the blood–brain barrier, and they are engulfed by cells through endocytosis or by direct fusion with the plasma membrane. For example, exosomes engineered to specifically target neurons by expressing rabies virus glycoprotein (RVG) via Lamp2b and loaded with miR124 delivered neuroprotection by increasing cortical neurogenesis when injected one-day post-ischemia in a murine model of stroke [149]. Moreover, exosomes derived from MSCs expressing miR133 were observed to be taken up by neurons and astrocytes when delivered through the tail vein 24 h after stroke. Treated animals with overexpressing miR133 exosomes showed significant neurite remodeling and functional recovery [119].

Interestingly, exosomes can do more than to deliver miRNA. It has been shown that MSCs-derived exosomes loaded with the anti-inflammatory compound curcumin—delivered twice a day intranasally for seven days after ischemic stroke—showed neuroprotection [150].

## 8. Conclusions

In this paper, we focused on neuronal cell death and the consequences of synaptic loss and EV release in stroke. It is clear that much research can be done to understand the basic mechanisms related to synaptic loss and, significantly, in brain communication with EVs after ischemic injury. This will not only increase our knowledge of underlying processes in stroke but also lead to the discovery of new potential therapeutic targets.
